# Thermodynamic, electrochemical and surface characterization of copper corrosion inhibition in acidic solution using rice straw extract

**DOI:** 10.1038/s41598-025-12482-w

**Published:** 2025-07-30

**Authors:** O. Adel, M. E. Mohamed, E. Khamis

**Affiliations:** 1 Chemistry Department, Faculty of Science, Alexandria University, Alexandria, Egypt; 2 Faculty of Advanced Basic Sciences, Alamein International University, Alamein City, Matrouh Governorate Egypt; 3 Science & Innovation Center of Excellence, SICE, Egyptian Russian University, Badr, Egypt

**Keywords:** Sustainability, Corrosion inhibition, Eco-friendly, Copper, Acidic, Biomass waste, Structural materials, Chemical engineering

## Abstract

The use of natural inhibitors offers a sustainable and cost-effective approach to mitigating copper corrosion. In this study, rice straw extract, an agricultural byproduct, was investigated as an eco-friendly corrosion inhibitor for copper in 0.5 M H_2_SO_4_ solutions. The corrosion inhibition performance was evaluated using electrochemical impedance spectroscopy (EIS) and potentiodynamic polarization techniques across a temperature range of 25–55 °C. The results demonstrated that the rice straw extract achieved an inhibition efficiency of approximately 91% at 25 °C with a concentration of 0.05 g/L and maintained a significant 69.1% efficiency at 55 °C, suggesting that the inhibition primarily occurs through physisorption of the extract molecules onto the copper surface. Scanning electron microscopy (SEM) analysis further confirmed the protective layer formation, while potential of zero charge (PZC) measurements indicated a negatively charged copper surface in sulfuric acid (− 13 mV). Adsorption isotherm studies revealed a strong binding constant, confirming that rice straw extract is an effective and sustainable corrosion inhibitor for copper in acidic environments.

## Introduction

Copper possesses remarkable physical and mechanical properties, along with excellent electrical and thermal conductivity, and it exhibits significant resistance to corrosion in various environments. As a result, it is widely used in machinery and facilities across numerous industrial sectors. Its applications include electrical wiring, plumbing, construction, and several industrial uses, such as renewable energy, electronics, and transportation. Copper alloys are commonly used in power plant condensers, oil cooler, low-pressure heater heat exchangers, and air-core conductors for water-cooled generators^1,2^. In the maritime industry, certain copper alloys are utilized in the military vessels’ water piping systems. However, the use of copper materials is often complicated by significant corrosion issues. The surface of copper and its alloys tends to oxidize, forming a fairly dense oxide film primarily composed of Cu(OH)_2_ and CuO being its principal constituents^3^. It is well established that the medium significantly influences the mechanism of copper electrodeposition and dissolution. Extensive research has focused on the anodic dissolution of copper in sulphate solutions leading to the identification of several distinct dissolution mechanisms. The literature generally indicates that intermediate Cu^+^ compounds, adsorbed Cu(I) species, or Cu^+^ ions play a role in the processes of copper dissolution or deposition^4–6^.

Corrosion poses a major economic challenge worldwide, impacting various sectors involved in the production, processing, and transportation of goods and equipment. To quantify its impact, researchers from different countries have periodically conducted corrosion cost assessments. These studies consistently show that annual corrosion-related expenses account for approximately 1 to 5% of a nation’s gross national product (GNP). According to a more recent estimate by NACE International, the global cost of corrosion has reached an estimated US$2.5 trillion—equivalent to 3.4% of the global gross domestic product (GDP)^7^.

As a result, the electrical and thermal conductivity of copper alloys are significantly compromised, considering the equipment and severely restricting the application of copper products. Typically, pickling is an effective method for achieving a clean copper surface by efficiently removing oxide deposit. Sulfuric acid is the most commonly used pickling solution, although it also attacks and corrodes the metal surface^3,8^. Since 0.5 M H_2_SO_4_ effectively removes oxides, scale, and other impurities from the metal’s surface, it is frequently used in acid pickling procedures for copper^9^. This concentration ensures effective cleaning while lowering the possibility of over-etching or damaging the copper by achieving a suitable equilibrium between reactivity and control. The strong protonation capacity of sulfuric acid promotes a clean substrate required for later procedures like plating or soldering by aiding in the breakdown of metal oxides. The use of corrosion inhibitors in industrial settings, as well as in academic research and literature, is one of the most significant and practical approaches to regulate the process of metallic corrosion^10–12^. Due to their rapid effect, organic compounds containing heteroatoms such as nitrogen, phosphorous, oxygen, and sulfur are frequently used as corrosion inhibitors to control the corrosion of metal surfaces^13–15^.

Recently, researchers have focused on replacing synthetic organic inhibitors with those derived from natural materials. This shift is driven by the high cost and harm associated with synthetic inhibitors, whereas natural product extracts are inexpensive, renewable, and environmentally friendly anti-corrosive alternatives^16–22^. As the concept of environmental sustainability gains momentum, plant extract-based corrosion inhibitors are increasingly recognized for their eco-friendly and effective protective properties. Hu et al. investigated the corrosion inhibition performance of *Capsicum annuum* L. leaf extract (CALLE) using polarization curve analysis and electrochemical impedance spectroscopy (EIS). The inhibition efficiency increased significantly, reaching approximately 93.9% at a concentration of 500 mg/L^23^. Sun et al. evaluated the anticorrosive effect of honeysuckle extract (HE) on copper in 0.5 mol/L H_2_SO_4_ using various experimental techniques combined with theoretical analysis. The results showed that HE achieved a maximum corrosion inhibition efficiency (η) of nearly 90%, with the efficiency remaining stable across different temperatures^24^. Zhang et al. studied the corrosion inhibition effect of *Davidia involucrata* leaf extract (DLE) on copper in 0.5 mol/L H_2_SO_4_ using both experimental and theoretical approaches. Electrochemical measurements revealed that the maximum inhibition efficiency (η) reached 89%^25^. Al-Nami et al. examined the corrosion behavior of copper in 2.0 M HNO₃ in the presence of *Calotropis procera* (CP) extract as a green inhibitor using AC impedance spectroscopy (EIS), Tafel polarization (PP), electrochemical frequency modulation (EFM), and weight loss (WL) methods. The results revealed that CP extract provides effective corrosion protection, achieving an inhibition efficiency of 84.7% at a concentration of 300 ppm^26^. Zhang et al. investigated *Idesia polycarpa* Maxim fruit extract (IFE) as a natural green corrosion inhibitor for copper in 0.5 M H_2_SO_4_ solution. Electrochemical impedance spectroscopy (EIS) results showed that the inhibition efficiency reached up to 90.8% at 298 K^27^.

Rice is the world’s second-largest cereal crop after wheat, and its cultivation produces vast quantities of rice straw as agricultural residue. Despite its potential value, only around 20% of rice straw is utilized in industries such as bioethanol production, paper manufacturing, and fertilizers. The remaining majority is often burned or abandoned in fields, practices that contribute to serious environmental issues including air pollution—commonly referred to as the “Black Cloud”—and depletion of soil nutrients. In response to the growing global emphasis on sustainability, the use of agricultural waste as a source of green corrosion inhibitors has gained increasing attention^22^. Rice straw, being rich in organic and biodegradable compounds, presents a promising alternative to traditional synthetic corrosion inhibitors^21^, which are typically hazardous, non-renewable, and expensive. As an eco-friendly inhibitor, rice straw extract distinguishes itself from other cereal straws by its high silica and lignin content, which are effective in the corrosion inhibition of metals^28^. Utilizing rice straw extract for corrosion protection not only addresses waste management challenges but also promotes environmentally responsible practices aligned with the principles of green chemistry and the circular economy.

In this study, we present a novel investigation into the utilization of rice straw extract as a corrosion inhibitor for copper in H_2_SO_4_ solutions. The aim of this study is to investigate the inhibitory effect of rice straw extract on copper corrosion in corrosive solutions containing 0.5 M H_2_SO_4_ at temperatures ranging from 25 to 55 °C. This will be achieved using various techniques, including electrochemical measurements and surface analysis. The effectiveness of the rice straw extract in controlling corrosion was evaluated using electrochemical measurements such as polarization and impedance measurements. Additionally, the surface morphology of the copper samples was analyzed using scanning electron microscopy (SEM). Fourier transform infrared spectroscopy FTIR analysis to identify the functional groups present in the rice straw extract.

## Experimental studies

### Solutions preparation

The test solutions of 0.5 M H_2_SO_4_ were prepared from distilled water and analytical grade H_2_SO_4_. The rice straw used in this research was sourced from a private field in Alexandria Governorate, Egypt. The rice straw was collected in accordance with institutional, national, and international guidelines and legislation. Ethanolic extracts of rice straw stock solution were obtained following the previously reported procedure^17^. Each test solution contains 10% ethanol.

**Fig. 1:**
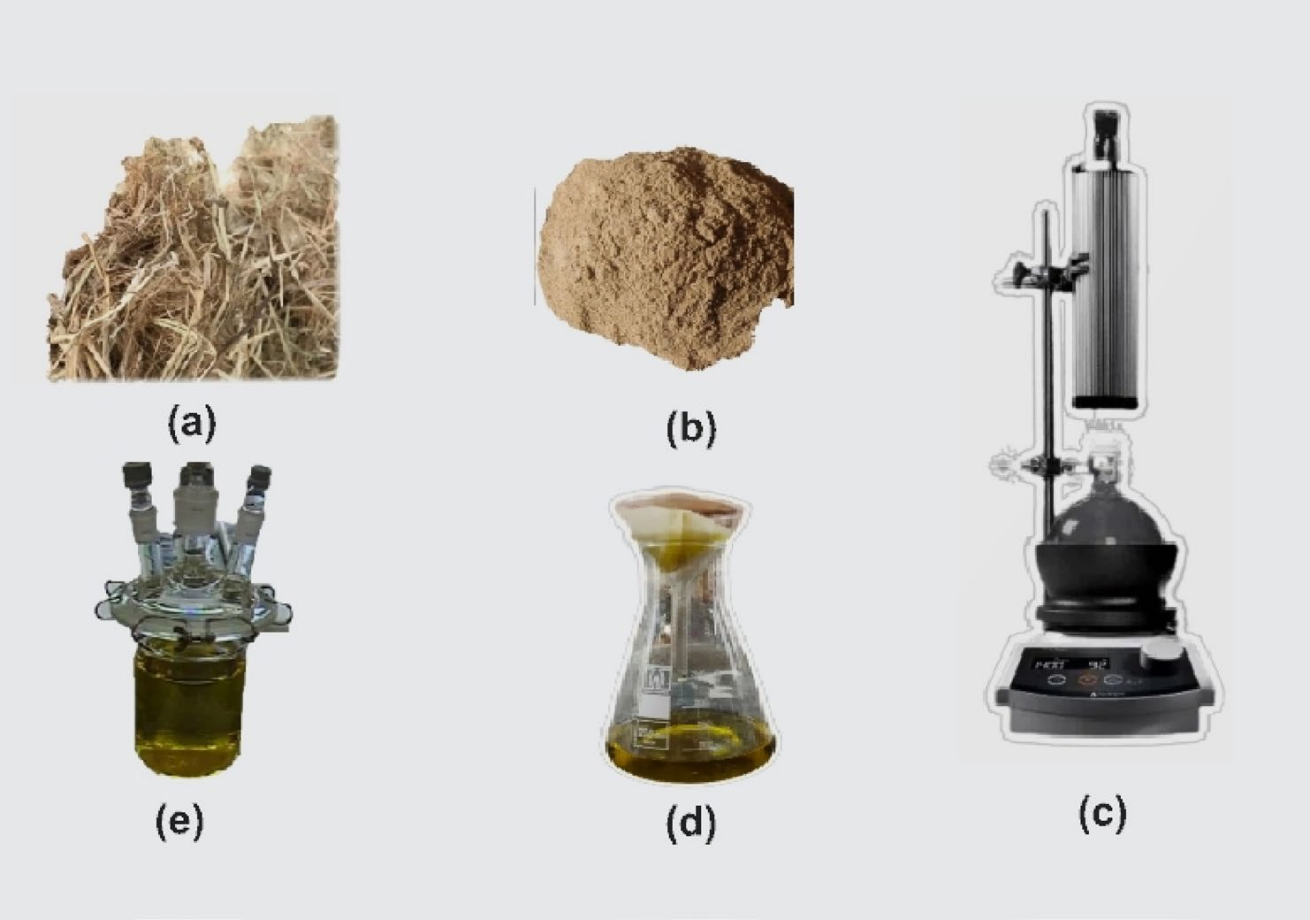
Schematic representation of the rice straw extraction process: (**a**) raw rice straw after cleaning and drying; (**b)** powdered rice straw obtained by grinding; (**c**) reflux extraction apparatus used for solvent extraction of bioactive compounds; (**d**) crude rice straw extract collected after filtration; (**e**) final filtered extract prepared for corrosion inhibition studies.

Figure 1 shows a schematic illustration for detailed description of the rice straw extraction process to visually represent the key steps.

As shown in the figure:

- represents the raw rice straw, collected, cleaned, and dried to remove impurities.
- shows the ground rice straw powder, prepared by milling the dried straw to a fine consistency to increase the surface area for extraction.
- illustrates the reflux extraction setup, where the powdered rice straw is subjected to solvent extraction (10% ethanol) under controlled temperature and stirring conditions to efficiently extract the bioactive compounds.
- displays the crude rice straw extract after filtration, containing the organic constituents responsible for corrosion inhibition.
- shows the final filtered extract ready for use in the corrosion inhibition tests.

This schematic representation, along with the textual description, clarifies the preparation methodology of the rice straw extract and ensures transparency and reproducibility of the experimental procedures.

### Characterization techniques

FTIR analysis of the rice straw extract was conducted using a Shimadzu FTIR 8400S, covering the spectral range from 4000 to 400 cm^–1^.

Scanning Electron Microscopy (SEM; model: JSM-200 IT, JEOL) analysis was performed using an accelerating voltage of 20 kV, under high-vacuum conditions to examine the surface morphology of the copper samples before and after immersion in 0.5 M H_2_SO_4_ solution with and without the rice straw extract. The copper samples were polished sequentially with emery papers ranging from 120 to 1000 grit to obtain a clean surface suitable for testing. Both in the presence and absence of the rice straw extract, abraded copper samples were immersed in a 0.5 M H_2_SO_4_ acid solution for 6 h. Subsequently, the samples of copper were allowed to dry at room temperature.

### Electrochemical tests

The frequency response analyzer (FRA)/potentiostat, ACM instrument (UK) was used to evaluate potentiodynamic polarization and electrochemical impedance spectroscopy (EIS) curves. The experimental settings and cell configuration matched the previously stated ones^15^. The working electrode was copper, with a composition 99.5% Cu and 0.5% Ca. A graphite rod was used as the counter electrode, while Standard Calomel Electrode (SCE) functioned as the reference electrodes. A cylindrical copper plate was encapsulated in Teflon, ensuring that only one side with a constant surface area of 0.2827 cm^2^ remained exposed to the solution. Before each experiment, the electrode surface was wetted and mechanically abraded using a succession of different-grade emery sheets, initially using coarse (120 grit) and working up to fine (1000 grit). The electrode was then thoroughly rinsed with double-distilled water and finally dried with absolute ethanol just prior to immersion in the testing solutions. Each experiment utilized a freshly polished electrode.

## Results and discussion

### FTIR measurements

**Fig. 2:**
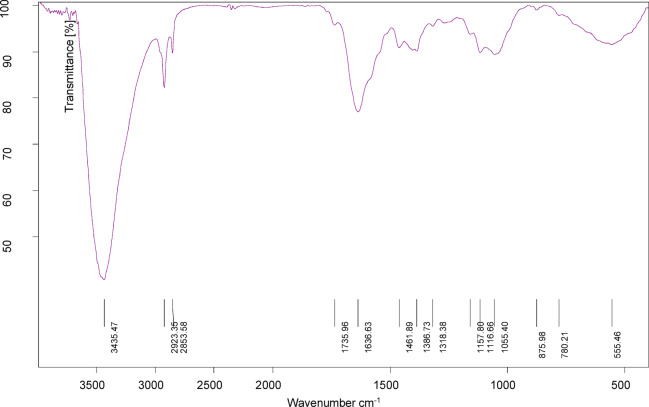
FTIR spectra of rice straw extract.

The FTIR measurements were conducted to characterize the functional groups present in the rice straw extract. Table 1 and Fig. 2 demonstrate FTIR spectrum bands and the band assignments for rice straw extract. Notably, a peak of absorption at 3435 cm^−1^ corresponds to the absorption bands for OH groups of the phenolic compound^29^. The C–H stretching vibration for aromatic ring’s is observed at 2923 cm^−1^, while absorption peaks at 2853 and 1735 cm^−1^ are attributed to the CH_2_ symmetric stretching vibration, and the stretching vibration of C=O occurs, respectively. Additionally, stretching vibrations for = C–H appear at 1636 cm^−1^^29,30^. These bands indicative of lignin, a chemicals extracted from rice straw^21^. The peak at 1461 cm^−1^ is associated with the aromatic C=C bond, an unsymmetric stretching of the C–O–C bridge bond and C–O–C cyclic ether is observed at 1157 and 1116 cm^−1^, respectively^31^.

**Table 1:** FTIR band assignments for rice straw extract.

Band, cm^−1^	Assignment
3435	OH of the phenolic group
2923	Absorption bands for C–H
2853	CH_2_ symmetric stretching vibration
1735	C=O stretching
1636	Stretching vibrations for = C–H
1461	Aromatic –C=C stretching vibrations
1386	COO^−^ symmetric stretch
1318	C–H deformation
1157	The C–O–C bridge unsymmetric stretching
1116	C–O–C Cyclic ether
1055	C–O stretching
875	Aromatic C–H bending

### Electrochemical measurements

#### Open–circuit potential (OCP) measurements

**Fig. 3:**
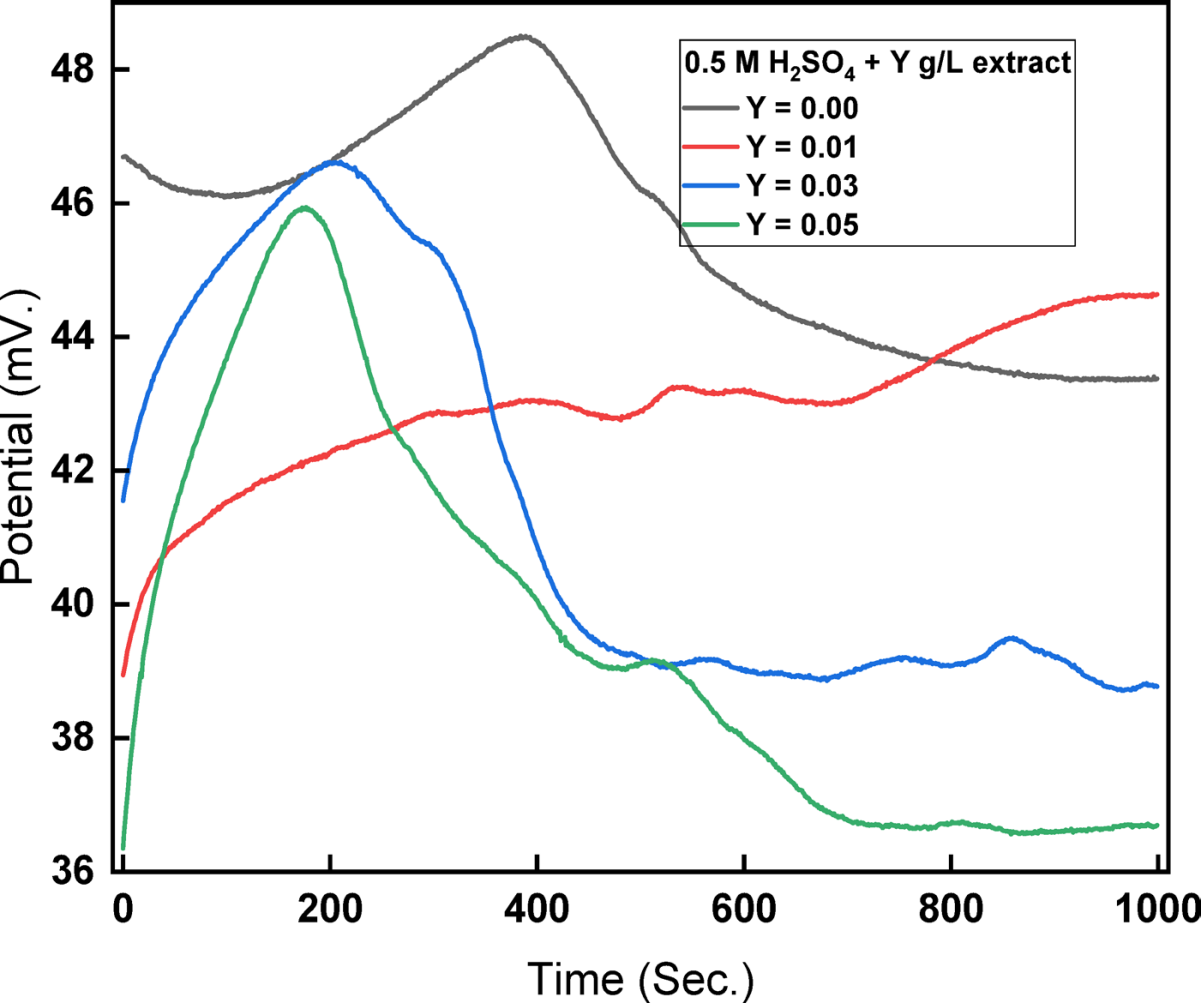
Potential-time curves for copper in 0.5 M H_2_SO_4_ at different concentrations of rice straw extract. The symbol Y represents the extract concentration.

OCP explains how the potential of copper electrodes in testing media with and without inhibitors relates to time until it stabilizes (reaches constant values). Figure 3 shows the potential-time curves for copper exposed to 0.5 M H_2_SO_4_ at different concentrations of rice straw extract as an inhibitor. After 16 min, which is thought to be enough time to establish an electrode equilibrium or quasi-equilibrium condition, experimental measurements were conducted. Because of the adsorption of organic molecules on the electrode surface, the electrode potential shifts to more negative values when varying amounts of rice straw are present.

#### Potentiodynamic polarization curves measurements

**Fig. 4:**
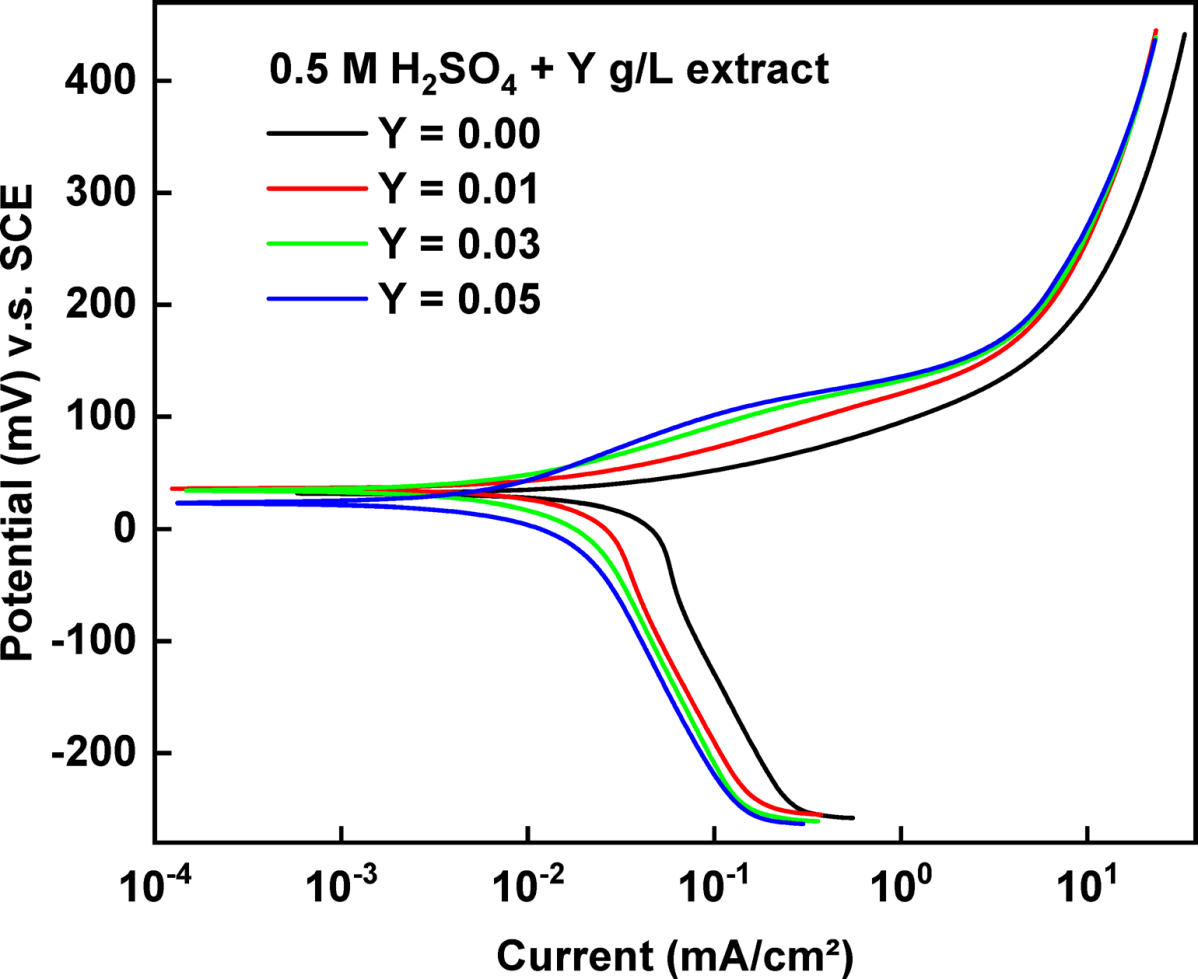
The electrochemical polarization curve for the corrosion of copper in 0.5 M H_2_SO_4_ containing different concentrations of rice straw extract, at 25 °C.

Figure 4 illustrates that increasing the concentration of rice straw extract suppresses both the anodic metal dissolution and the cathodic reduction of dissolved oxygen reactions, indicating that rice straw acts as a mixed-type inhibitor. As well as, the corrosion current density (i_corr_) values decrease while the inhibition efficiency %η increases. The anodic curve displays two distinct regions: the active dissolution (apparent Tafel region) and the limiting current zone, which are characteristic of copper in H_2_SO_4_ solution. The mechanism underlying these observations is detailed in reference^32^.

The first region corresponds to the active dissolution of copper, which can be represented by the following two continuous step’s reactions:

1 $${\text{Cu}}_{{({\text{S}})}} \to {\text{ Cu}}^{ + }_{{({\text{ads}})}} + {\text{ 1e}}^{ - } \left( {{\text{fast}}} \right)$$

2 $${\text{Cu}}^{ + }_{{({\text{ads}})}} \to {\text{ Cu}}^{{{2} + }}_{{({\text{sol}})}} + {\text{ 1e}}^{ - } \left( {{\text{slow}}} \right)$$

where Cu^+^_(ads)_ is not diffused into the main solution, but is instead an adsorbed species on the copper surface. However, equilibrium suggests that trace amounts of Cu^+^_(sol)_ can be detected in the solution, as noted in reference ^33^.

3 $${\text{Cu}}^{{{2} + }}_{{({\text{sol}})}} + {\text{ Cu}}_{{({\text{S}})}} \leftrightarrow {\text{ 2 Cu}}^{ + }_{{({\text{sol}})}}$$

The second region is the passivation region, during which a passive layer of Cu_2_O film is formed on the surface. This layer acts as a barrier, blocking the copper surface from the aggressive medium and consequently reducing copper corrosion^34^. The reaction can be represented as follows^35^:

4 $${\text{Cu}}_{{({\text{S}})}} + {\text{ H}}_{{2}} {\text{SO}}_{{{4}({\text{L}})}} \to {\text{ Cu}}_{{2}} {\text{O}}^{{}}_{{({\text{S}})}} + {\text{ SO}}_{{{2}({\text{g}})}} + {\text{ H}}_{{2}} {\text{O}}_{{({\text{L}})}}$$

The obtained electrochemical polarization parameters are listed in Table 2. The percentage inhibition efficiency (% η) was calculated using the equation:

5 $$\% \upeta = \left[ {\left( {{\text{i}}_{{\text{o}}} - {\text{i}}} \right)/{\text{i}}_{{\text{o}}} } \right] \times {1}00$$

where i_o_ and i are the corrosion current densities in the absence and presence of extract.

**Table 2:** The electrochemical polarization parameters for the corrosion of copper in 0.5 M H_2_SO_4_ containing different concentrations of rice straw extract, at 25 °C.

[Extract], g/L	E_corr_ (mV)	i_corr_ (µA cm^−2^)	β_a_ mV/decade	−β_C_ mV/decade	% η ± SD
0.00	18.2	0.043852	56.4	405.9	–
0.01	36.4	0.023341	52.5	424.9	46.8 ± 1.07
0.02	42.4	0.012612	49.6	278.9	71.2 ± 1.25
0.03	45.4	0.011294	48.2	196.0	74.2 ± 2.00
0.04	45.4	0.010008	51.6	220.3	77.2 ± 1.53
0.05	43.0	0.008385	52.8	172.6	80.9 ± 2.00

As shown in Table 2, the Tafel slope values change noticeably with increasing concentration of rice straw extract. The anodic slope (β_a_) remains relatively stable, with only slight fluctuations, suggesting that the rice straw extract does not significantly alter the anodic dissolution mechanism of copper. However, the cathodic slope (−β_c_) shows a marked decrease from 405.9 mV/decade at 0 g/L to 172.6 mV/decade at 0.05 g/L. This trend indicates a change in the cathodic reaction kinetics, likely due to the adsorption of extract molecules on active cathodic sites, which impedes the hydrogen evolution reaction. The decreasing β_c_ values imply that the rice straw extract acts more strongly on the cathodic reaction, reducing its rate by forming a protective film that blocks proton access to the surface. This supports the conclusion that the extract functions as a mixed-type inhibitor, with predominant cathodic inhibition behavior. These observations are consistent with the corresponding increase in inhibition efficiency and confirm that the extract effectively modifies the electrochemical processes occurring on the copper surface.

This is attributed to the presence of functional groups such as hydroxyl (–OH), methoxyl (–OCH_3_), methyl (CH_3_), and aromatic rings can effectively slow down the corrosion process. Also, organic substances featuring electron-donating groups, such as hydroxyl (–OH), methoxyl (–OCH_3_), and methyl (CH_3_), are recognized as effective corrosion inhibitors for metals in acidic environments.

The comparison presented in Table 3 demonstrates the effectiveness of rice straw extract as a corrosion inhibitor relative to several other plant-based systems reported in recent literature. While many natural extracts achieve high inhibition efficiencies in acidic media, they often require relatively high concentrations (typically 300–500 mg/L) to be effective. In contrast, the rice straw extract used in this study achieved a comparable or superior inhibition efficiency of 91.39% at just 50 mg/L, highlighting its potency even at low dosages. This not only reflects the strong adsorption capability of the extract’s active components on the copper surface but also underscores the potential synergistic effect of the various organic constituents naturally present in rice straw. Additionally, rice straw offers the added advantage of being an abundant and low-cost agricultural waste material, making it a more sustainable and economically viable option. These findings emphasize the dual benefits of the extract—environmental friendliness and high corrosion inhibition performance—making it a promising candidate for green corrosion control in industrial applications.

**Table 3:** comparative performance of rice straw extract with other plant-based corrosion inhibitors for copper in acidic media.

Extract name	Optimal concentration	Metal base	Medium	Anti-corrosion efficiency (%)	Year
*Capsicum Annuum* L. ^23^	500 mg/L	Copper	0.5 M H_2_SO_4_	93.9	2024
*Honeysuckle*^24^	400 mg/L	Copper	0.5 M H_2_SO_4_	90.0	2023
*Davidia Involucrata Leaf*^25^	300 mg/L	Copper	0.5 M H_2_SO_4_	89.0	2021
*Idesia Polycarpa Maxim Fruit*^27^	300 mg/L	Copper	0.5 M H_2_SO_4_	90.8	2020
*Calotropis Procera*^26^	300 mg/L	Copper	2 M HNO_3_	84.7	2019
*Rice Straw (Present Study)*	50 mg/L (0.05 g/L)	Copper	0.5 M H_2_SO_4_	91.39	Present

#### Electrochemical impedance spectroscopy (EIS) measurements

**Fig. 5:**
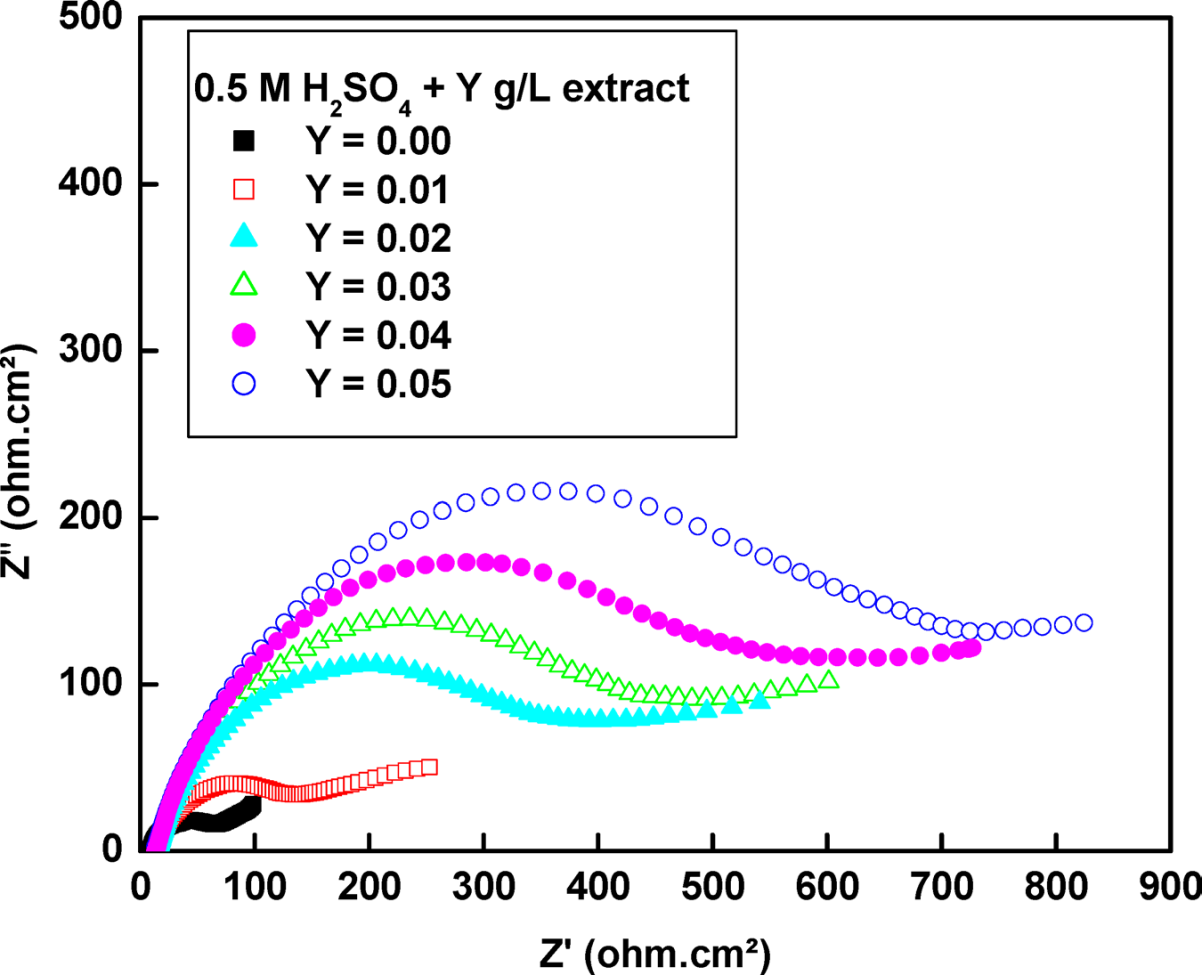
The electrochemical impedance curve for the corrosion of copper in 0.5 M H_2_SO_4_ containing different concentrations of rice straw extract at 25 °C.

As seen in Fig. 5, the **Nyquist** plots characterized at the low frequency zone by a straight line (exhibits Warburg impedance) and at the high frequency zone with a capacitive reactance arc which is commonly connected to charge transfer resistance and the electric double layer capacitance. The presence of the low-frequency line indicates that the copper is undergoing corrosion in 0.5 M H_2_SO_4_.

The straight line at the low frequency region disappears as the concentration of rice straw increases, indicating that the corrosion product on the copper surface is successfully diffusing into the bulk solution. Additionally, the noticeable increase in the capacitive reactance arc’s radius corresponds to a rise in the charge transfer resistance at the surface of the copper electrode. This implies that rice straw forms a thick resistant film on the copper surface, which reduces corrosion effectively. Furthermore, the capacitive reactance arc appears as imperfect semicircles, reflecting the heterogeneity of the copper surface.

**Fig. 6:**
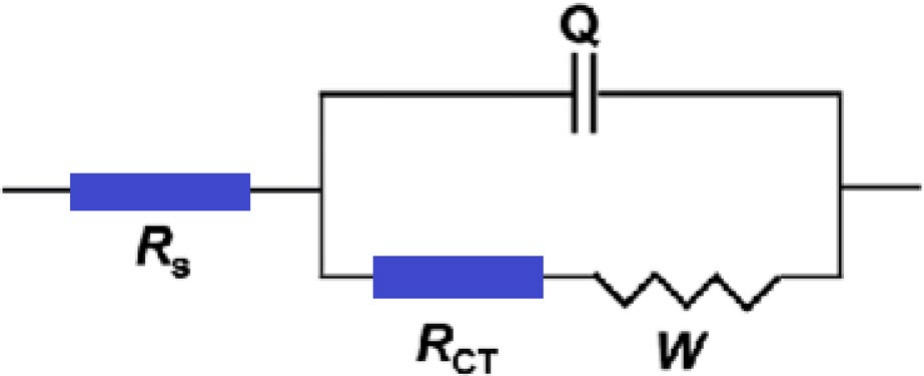
The equivalent circuit model used to fit the EIS experiment.

These plots were examined by adjusting the experimental data to the corresponding circuit model displayed in Fig. 6 using the Zsimpwin program.

**Fig. 7:**
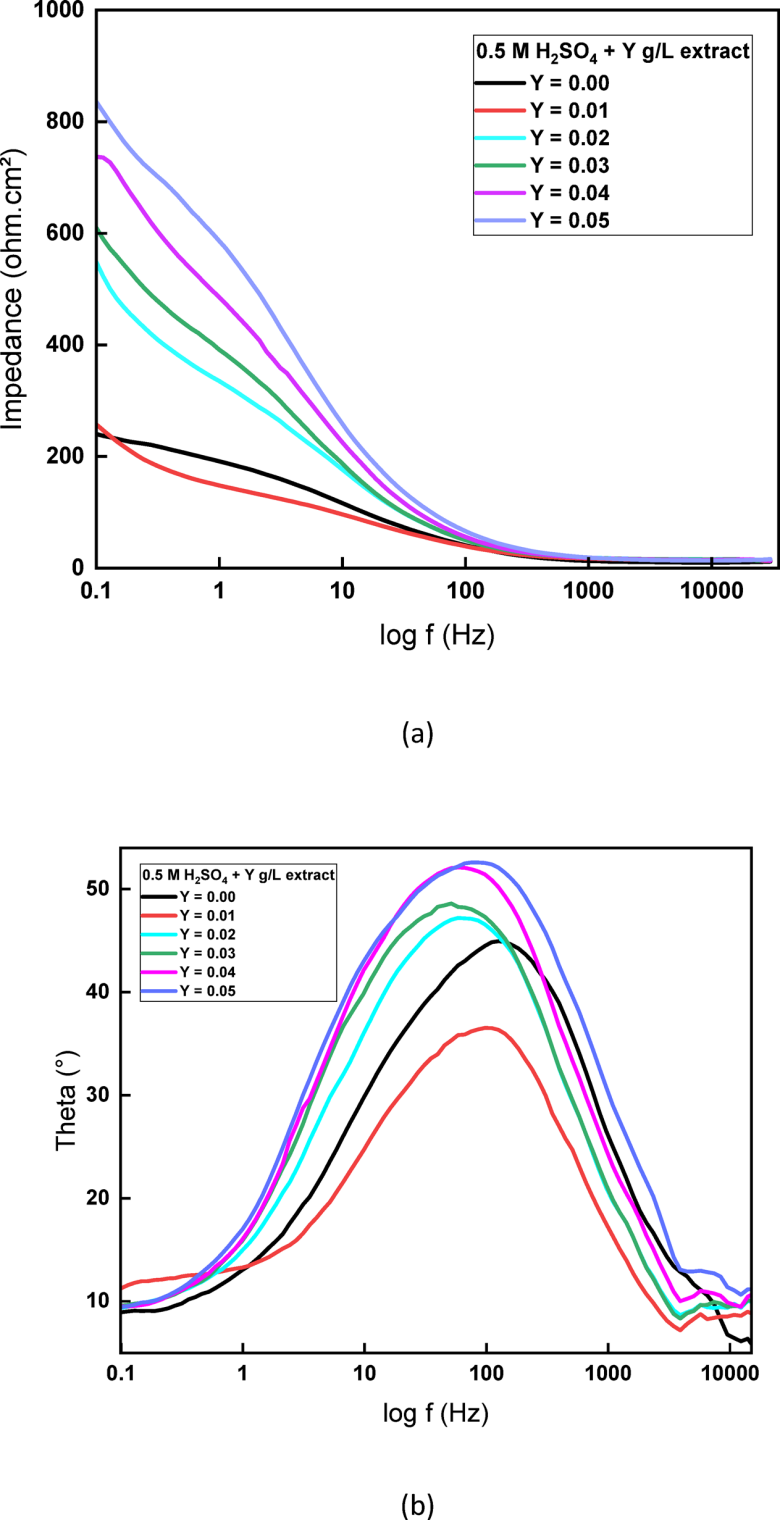
Bode plots of Cu in 0.5 M sulfuric acid with different concentrations of rice straw extract at 298 K.

The Bode plots in Fig. 7 illustrate the electrochemical behavior of copper in 0.5 M H_2_SO_4_ in the absence and presence of rice straw extract at various concentrations (0.01–0.05 g/L). In the impedance magnitude plot, the bare copper sample (Y = 0.00 g/L) exhibits the lowest impedance across the frequency spectrum, indicating low resistance to charge transfer and high corrosion susceptibility. With increasing inhibitor concentration, impedance values rise significantly, reflecting improved corrosion resistance due to the formation of a protective film. At the highest concentration (0.05 g/L), the impedance exceeds 800 Ω·cm^2^, confirming the formation of a strong barrier layer that impedes ionic transport at the copper–electrolyte interface. This trend supports the adsorption of extract molecules onto the copper surface, blocking active corrosion sites. Complementary to these findings, the phase angle (θ–log f) plot shows a noticeable shift in the maximum phase angle toward higher values and broader frequency ranges as the inhibitor concentration increases, reaching a peak of approximately 50° at 0.05 g/L. This indicates enhanced capacitive behavior and a more uniform inhibitor film, which collectively reduces the corrosion rate. The phase angle broadening and frequency shift further implies a slower electrochemical process and better surface coverage, both characteristics of an effective mixed-type inhibitor. These observations are consistent with the EIS fitting results based on an equivalent circuit model incorporating Warburg impedance, which captures the diffusion-controlled behavior of the system. The increase in charge transfer resistance (R_ct_) and decrease in double-layer capacitance (C_dl_) observed from the model fitting support the conclusion that rice straw extract forms a stable, adsorbed film that hinders both anodic and cathodic reactions, confirming its efficacy as a green corrosion inhibitor.

The impedance data were fitted using an equivalent circuit model of the form R[RW.Q], which effectively represents the electrochemical behavior of the copper surface in 0.5 M H_2_SO_4_. This model includes:

R_s_: Solution resistance, representing the ohmic resistance of the bulk electrolyte.

R_ct_: Charge transfer resistance, representing the resistance to the electron transfer process.

W: Warburg impedance, accounting for ion diffusion to and from the electrode surface, particularly significant at low frequencies.

Q (CPE): A constant phase element used in place of an ideal capacitor to better represent the non-ideal capacitive behavior of the interface. The use of a CPE reflects surface heterogeneity and roughness often present in real corrosion systems.

**Fig. 8:**
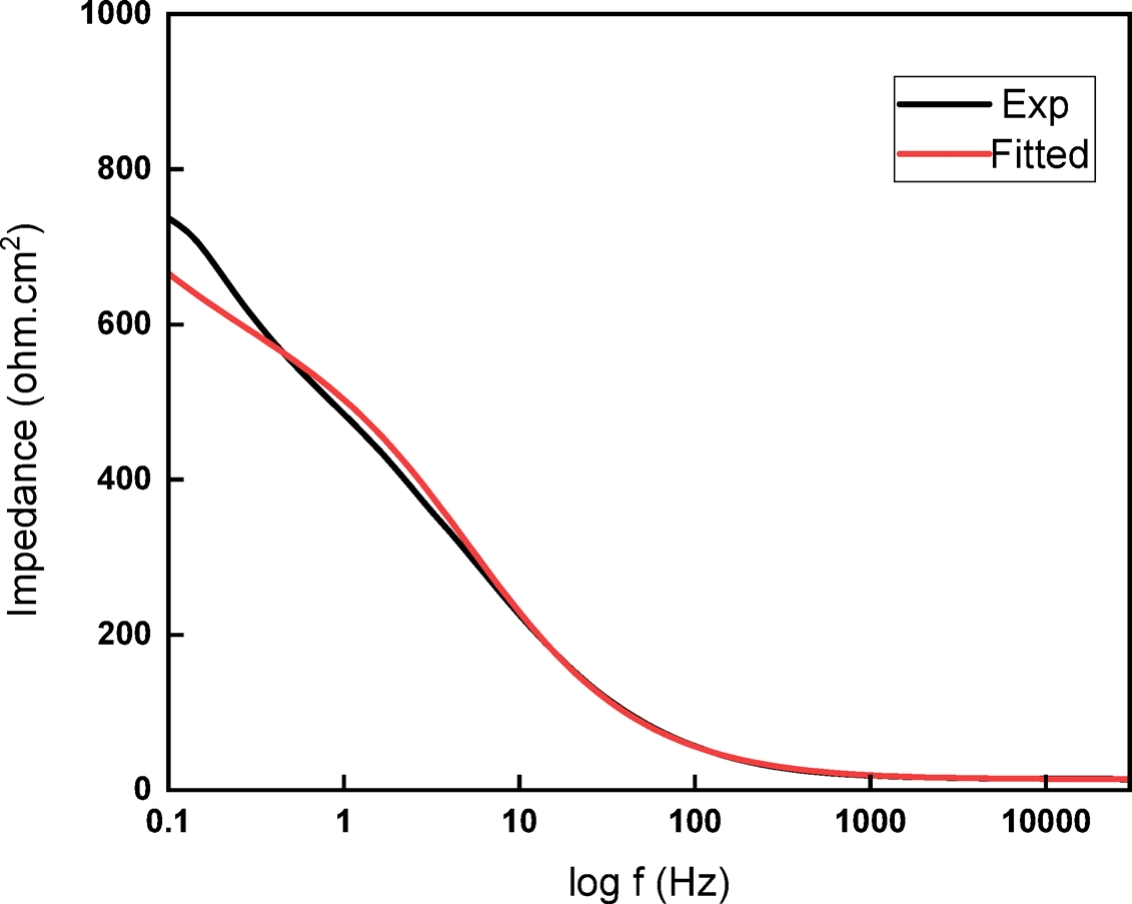
Experimental and fitted EIS data for copper in 0.5 M H_2_SO_4_ with 0.04 g/L rice straw extract at 25 °C.

As shown in Fig. 8 that there is an excellent agreement between the experimental and fitted curves; especially in the low-frequency region, which confirms the reliability and validity of the chosen R[RW.Q] model. This alignment demonstrates that the circuit adequately captures both the diffusion-controlled behavior (via Warburg impedance) and the non-ideal capacitive response (via the CPE), which are directly linked to the chemical-surface state of the copper in the presence of the rice straw extract.

In a non-homogeneous system, capacitances are replaced with a constant phase element (Q), characterized by the values of Q and n. The ideal double-layer capacitance (C_dl_) was derived using the equation^36^:

6 $${\text{C}}_{{{\text{dl}}}} = \, \left( {{\text{y}}_{0} \times {\text{ R}}_{{{\text{ct}}}} } \right)^{{{1}/{\text{n}}}} /{\text{R}}_{{{\text{ct}}}}$$

where y_0_ refers to the admittance of the constant phase element (CPE), n is the deviation parameter (− 1 ≤ n ≤ 1). A value of n = 1 indicates an ideal capacitor, while n = 0.5 implies Warburg-type behavior, reflecting diffusion effects^37^. The non-ideal nature of the surface, as shown by the deviation of n from unity, supports the physical interpretation of a partially inhibited surface with adsorbed organic compounds forming a protective, but non-uniform, barrier.

The consistent and close fit of the data not only validates the selected model but also provides insight into the surface condition and interaction between the inhibitor molecules and the copper substrate.

The data in Table 4 reveals that the C_dl_ is not a pure capacitor because the value of n is less than 1.

**Table 4:** The electrochemical impedance parameters for the corrosion of copper in 0.5 M H_2_SO_4_ containing different concentrations of rice straw extract at 25 °C.

[Extract], g/L	R_s_ (Ω cm^2^)	R_ct_ (Ω cm^2^)	Q = Y_o_ × 10^–6^ µs^n^/Ω cm^2^	n	W, µs^5^/Ω cm^2^	C_dl_ × 10^–5^ (μF/Cm^2^)	% η ± SD
0.00	9.146	58.21	384.2	0.714	0.02486	8.3716	–
0.01	13.85	135.3	342.6	0.701	0.01316	9.2145	56.97 ± 1.50
0.02	14.53	361.7	204.1	0.734	0.00928	7.9374	83.90 ± 1.33
0.03	14.45	450.2	207.3	0.733	0.01035	8.7381	87.07 ± 1.42
0.04	13.87	556.8	158.4	0.752	0.00786	7.1057	89.54 ± 1.00
0.05	12.83	676.7	143.4	0.738	0.00835	6.2566	91.39 ± 1.16

The data implies that as the extract’s concentration increases, the charge transfer resistance, R_ct,_ also increases, leading to a corresponding rise in inhibition efficiency increases. The decrease in the double-layer capacitance (C_dl_) values is attributed to the adsorption of the chemical constituents of rice straw on the copper surface.

Table 4 shows the electrochemical impedance parameters obtained. The percentage inhibition efficiency (% η) was calculated using the following equation^38^:

7 $$\% \upeta = \left[ {\left( {R_{{{\text{ct}}}} {-}R^{{\text{o}}}_{{{\text{ct}}}} } \right)/R_{{{\text{ct}}}} } \right] \times {1}00$$

### Potential of zero charge

**Fig. 9:**
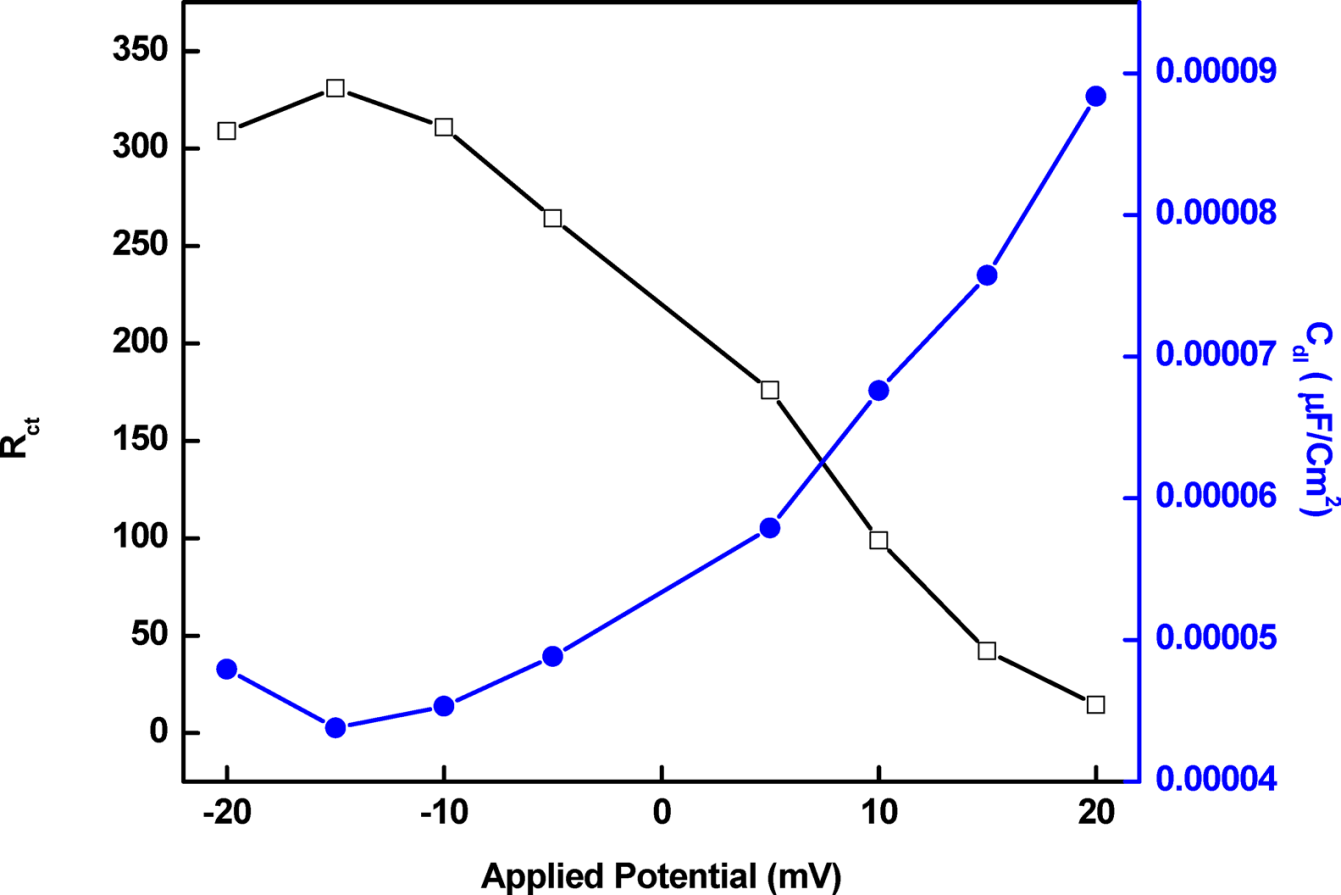
Variation of R_ct_ and C_dl_ with the applied potential for copper in 0.5 M sulfuric acid solution.

The potential of zero charges (PZC) is a crucial variable in electrostatic processes. Figure 9 illustrates the varying values of the double-layer capacitances as a function of applied voltage derived from the equivalent circuit previously discussed in Fig. 6. The minimum capacitance is attained at the PZC of a substance in solution. The surface charge of the metal is determined by the open circuit potential in relation to the PZC^31^. Copper in a solution of 0.5 M sulfuric acid has an open circuit potential (E_ocp_) and an E_PZC_ value of about − 28 mV and − 15 mV, respectively. Er is Antropov rational corrosion potential, and the equation Er = E_ocp_−E_pzc_ is used to calculate the surface charge (Er) of copper^39^. The findings demonstrate that the copper surface is negatively charged in 0.5 M sulfuric acid (− 13 mV).

### Adsorption considerations

**Fig. 10:**
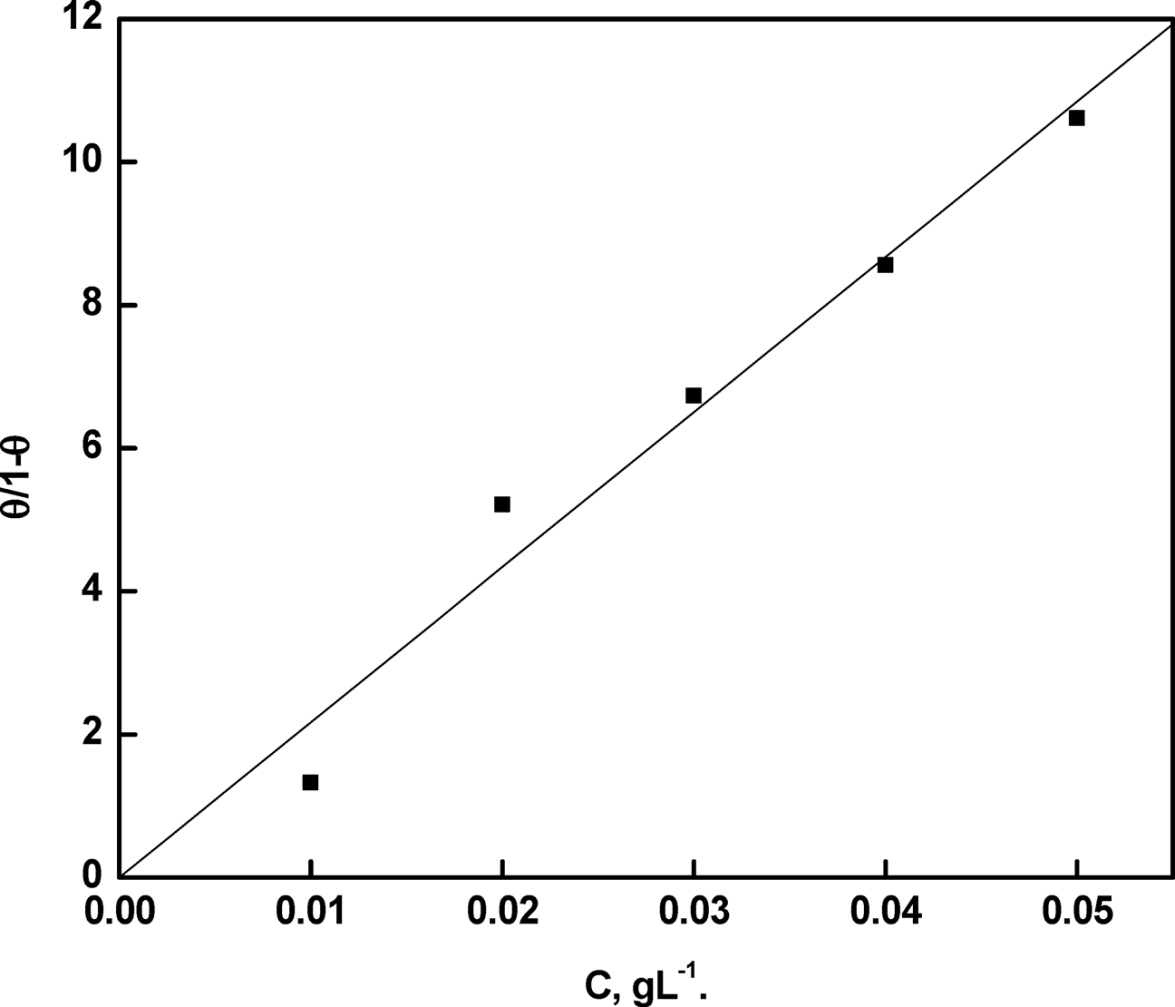
Linear fitting of the experimental data to Langmuir adsorption Isotherm for rice straw extract in 0.5 M H_2_SO_4_.

**Fig. 11:**
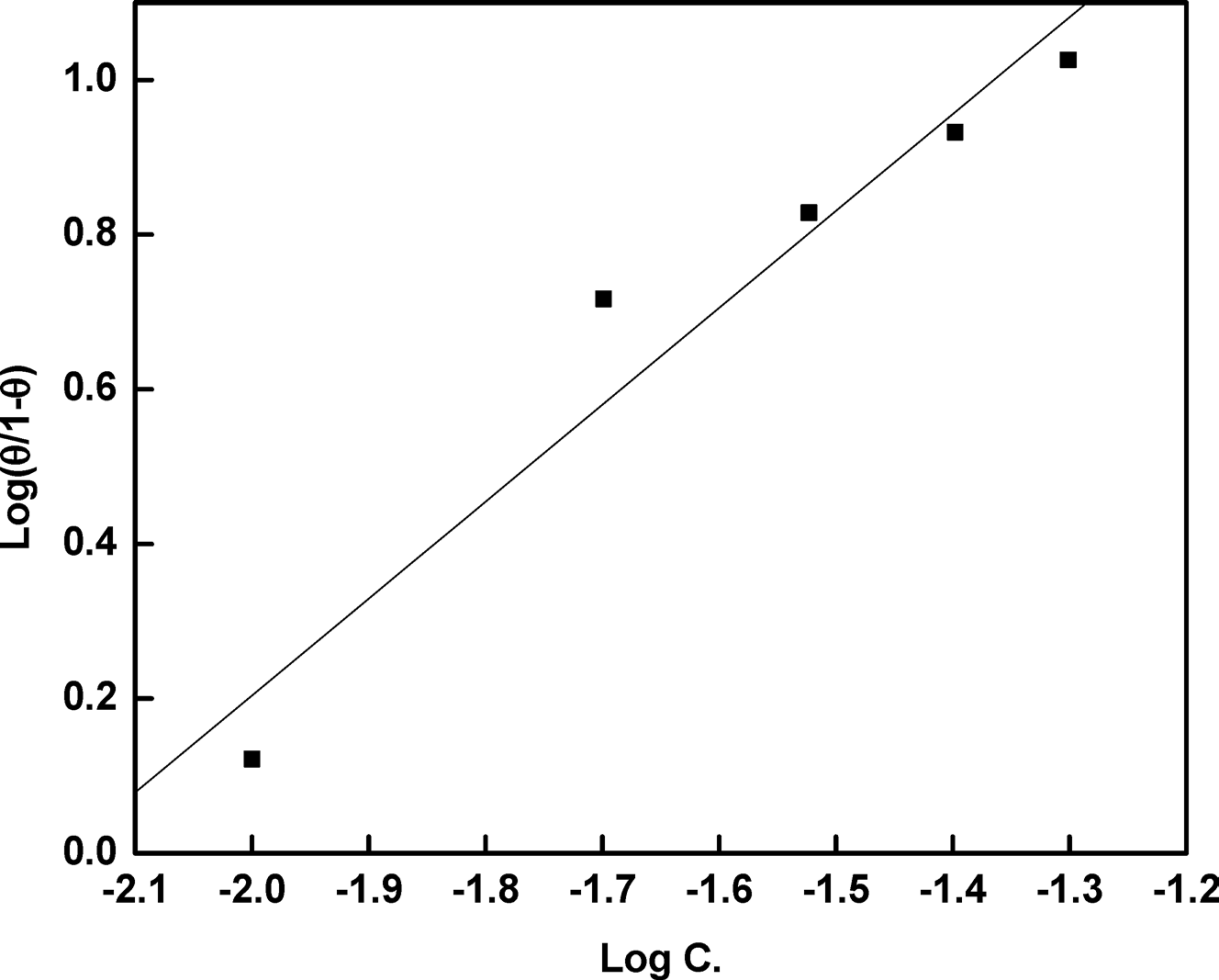
Linear fitting of the experimental data to Kinetic-thermodynamic model for rice straw extract in 0.5 M H_2_SO_4_.

Figures 10 and 11 present the theoretical fitting of the experimental data derived from the measurements of the EIS curves to the Langmuir adsorption isotherm as well as the Kinetic-Thermodynamic model^39–41^.

Table 5 presents the fitting parameters. According to Fig. 10, Langmuir adsorption isotherm most accurately describes the ideal adsorption behavior of rice straw extract on the copper surface, as it produces a straight line extending from the origin well with the experimental results.

**Table 5:** Adsorption parameters obtained from fitting of the experimental data to Langmuir and Kinetic-thermodynamics models for rice straw extract in 0.5 M H_2_SO_4_.

Langmuir		Kinetic-thermodynamics model		
K	R^2^	1/y	K	R^2^
216.8	0.968	0.797S	145.44	0.942

The equation can be represented as:

8 $$\frac{\uptheta }{1-\uptheta }={K}_{ads} \cdot C$$

where:

- C = concentration of the inhibitor in the solution
- θ = surface coverage (i.e., the fraction of the surface covered by the inhibitor) which can be estimated using the following formula: θ = %η/100
- K_ads_ = equilibrium constant of adsorption

Figure 11 demonstrates the applicability of the kinetic-thermodynamic adsorption model, which is expressed by the following equation:

9 $$\log \frac{\uptheta }{1 - \uptheta } = \log K^{\prime} + y \log C$$

where:

- θ = surface coverage
- C = concentration of the inhibitor
- K′ = the modified equilibrium constant related to the adsorption process
- y = the number of inhibitor molecules occupying active sites on the metal surface

The value of 1/y indicates the number of active sites covered by a single inhibitor molecule and helps assess the degree of surface interaction. A value of y close to 1 supports the ideal behavior of the adsorption process, suggesting monolayer adsorption. The magnitude of K′ reflects the strength of the interaction between rice straw extract molecules and the copper surface; a higher K′ value implies stronger bonding and greater adsorption affinity, whereas a lower value points to a weaker interaction. These results support the effective and spontaneous adsorption of rice straw extract components onto the copper surface^42^.

### Effect of temperature

The impact of temperature on copper corrosion in the sulfuric acid solution has been the subject of several studies. In this work, we examine the corrosion of copper in 0.5 M sulfuric acid at 25 °C, 35 °C, 45 °C, and 55 °C in the presence and absence of 0.03 g/L of rice straw extract.

**Fig. 12:**
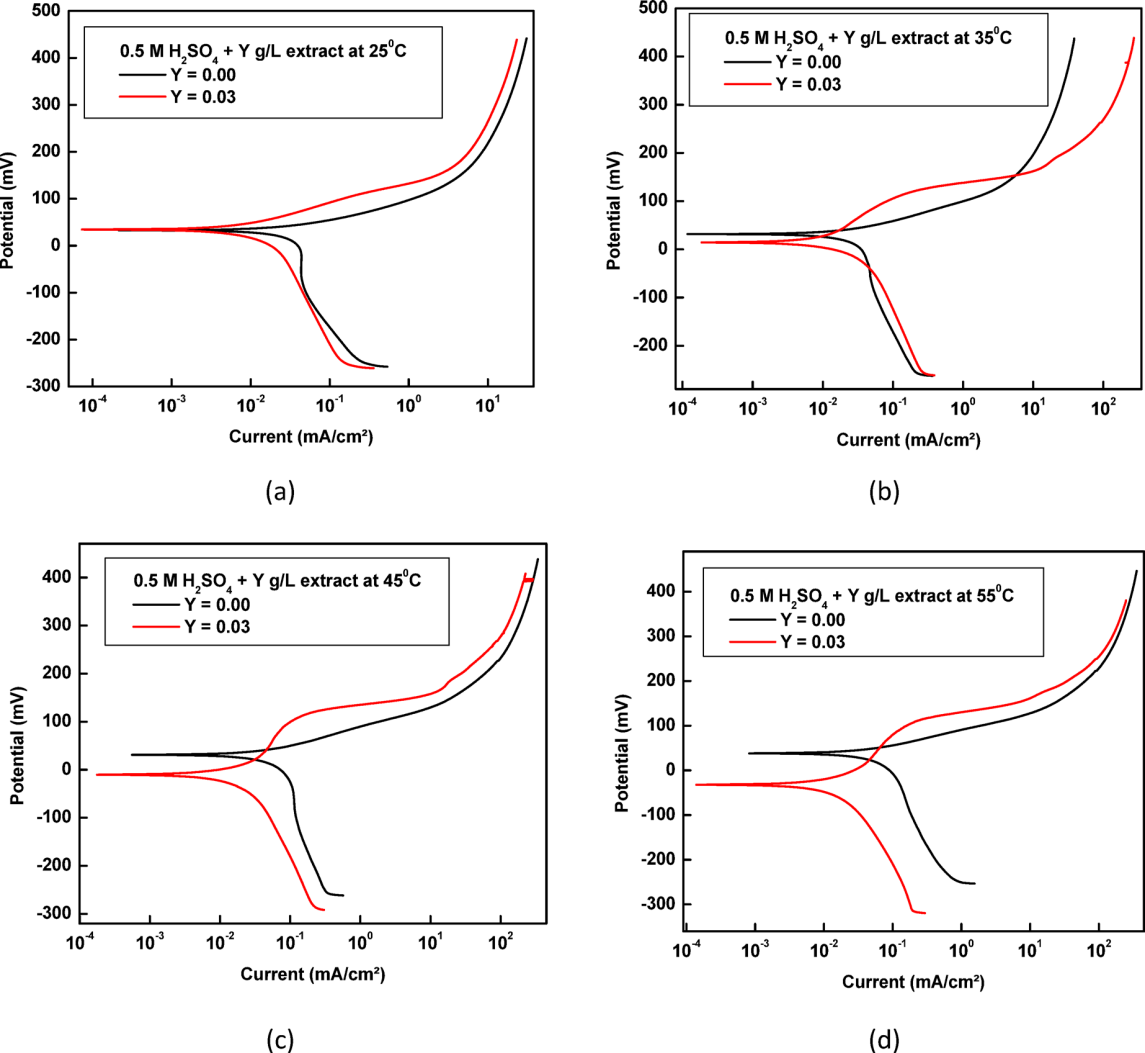
The electrochemical polarization curve for the corrosion of copper in 0.5 M H_2_SO_4_ containing rice straw extract, at different temperatures, where (**a**) at 25 °C, (**b**) at 35 °C, (**c**) at 45 °C, (**d**) at 55 °C.

The polarization curves presented in Fig. 12 demonstrate that at higher temperatures, the rice straw remains an effective inhibitor, with an inhibition efficiency (%η) of approximately 69.1% at 55 °C. Table 6 illustrates the electrochemical polarization parameters at different temperatures, both in the absence and presence of the rice straw extract at 0.03 g/L concentration.

**Table 6:** The electrochemical polarization parameters for the corrosion of copper in 0.5 M H_2_SO_4_ containing rice straw extract, at different temperatures.

°C	[Extract], g/L	E_corr_ (mV)	i_corr_ (µA cm^−2^)	β_a_ mV/decade	−β_C_ mV/decade	% η ± SD
25	0.00	18.2	0.043852	56.4	405.9	–
	0.03	45.4	0.011294	48.2	196.0	74.2 ± 1.20
35	0.00	34.1	0.033464	46.1	542.2	–
	0.03	35.3	0.012165	75.4	129.3	63.6 ± 1.49
45	0.00	42.4	0.058408	38.2	295.4	–
	0.03	− 31.1	0.022325	228.6	180.5	61.8 ± 1.14
55	0.00	49.4	0.048750	33.8	231.3	–
	0.03	− 50.2	0.017826	183.2	184.3	69.1 ± 1.50

These parameters—E_corr_ (corrosion potential), i_corr_ (corrosion current density), β_a_ (anodic Tafel slope), −β_c_ (cathodic Tafel slope), and %η (inhibition efficiency)—are key indicators of the corrosion behavior of copper and the efficiency of the inhibitor under various thermal conditions.

- A decrease in i_corr_ with inhibitor addition at all temperatures indicates effective corrosion suppression by the extract.
- The Tafel slopes (β_a_ and −β_c_) show notable variation, especially at elevated temperatures, reflecting changes in the kinetics of both anodic and cathodic reactions.
- The shift in E_corr_ in both positive and negative directions supports the classification of the rice straw extract as a mixed-type inhibitor.
- The inhibition efficiency (%η) generally decreases with rising temperature, suggesting partial desorption of inhibitor molecules at higher thermal energy, but still maintains significant protective ability.

These results collectively highlight the temperature-dependent behavior of the rice straw extract and support its role as an effective green corrosion inhibitor across a range of thermal conditions. The standard deviation (± 5.62) for %η values also confirms the reproducibility and reliability of the results.

The Arrhenius equation:

10 $${\text{ln k }} = \, - {\text{E}}_{{\text{a}}} /{\text{RT }} + {\text{ A}}$$

describes the linear relationship between the reciprocal of the absolute temperature, 1/T, and the logarithm of the corrosion rate (k). The rates of corrosion were expressed as the corrosion current density. Here, E_a_ represents the apparent effective activation energy, T denotes the absolute temperature, R is the universal gas constant, and A is the Arrhenius pre-exponential factor.

An alternative version of the Arrhenius equation is the transition state equation:

Where N is Avogadro’s number, h is Plank’s constant, ΔH* is the activation enthalpy, and ΔS* is the activation entropy, we get:

11 $${\text{k }} = \, \left( {{\text{RT}}/{\text{Nh}}} \right){\text{ exp }}\left( {\Delta {\text{S}}*/{\text{R}}} \right){\text{ exp }}\left( { - \Delta {\text{H}}* \, /{\text{RT}}} \right)$$

**Fig. 13:**
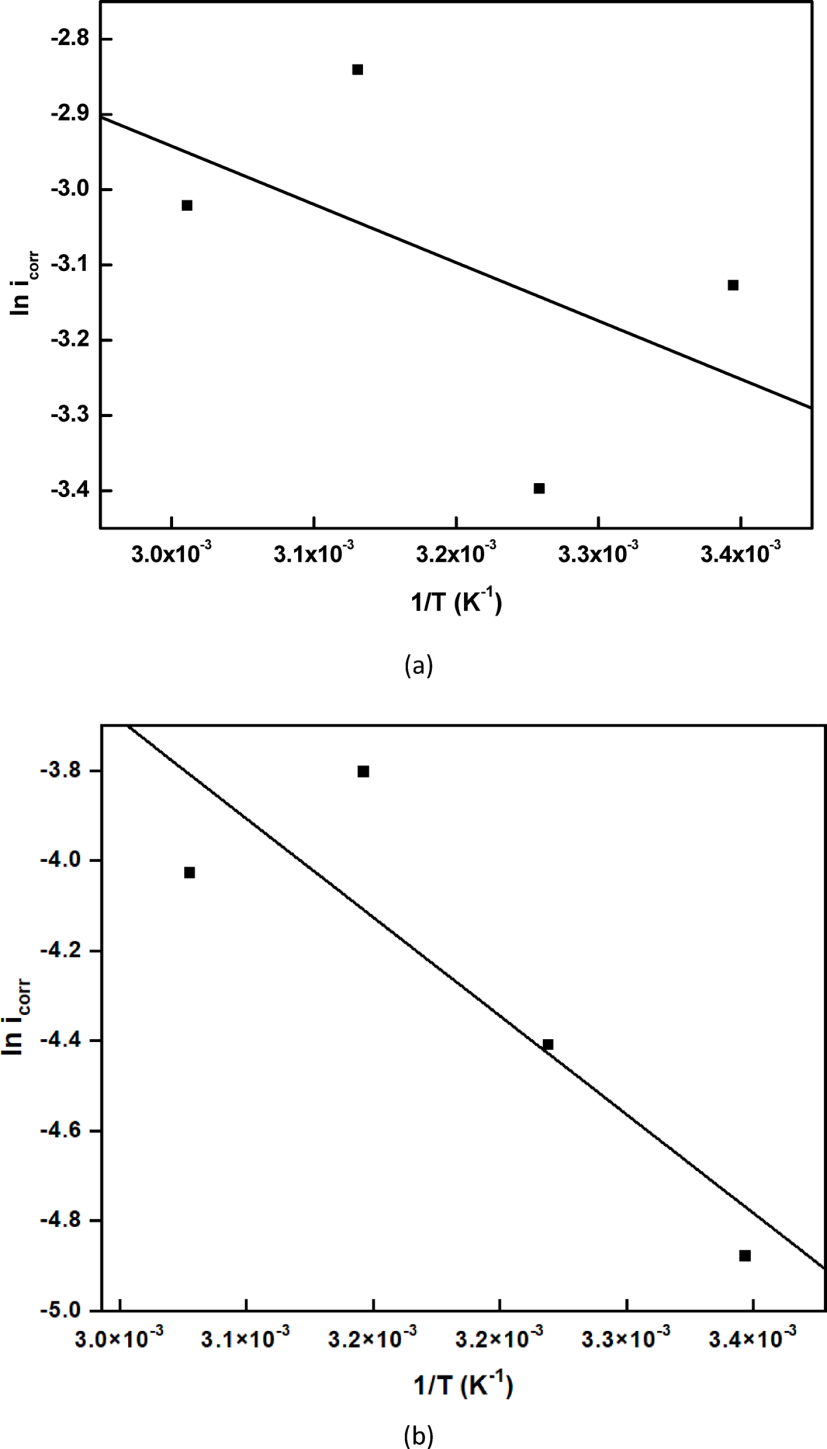

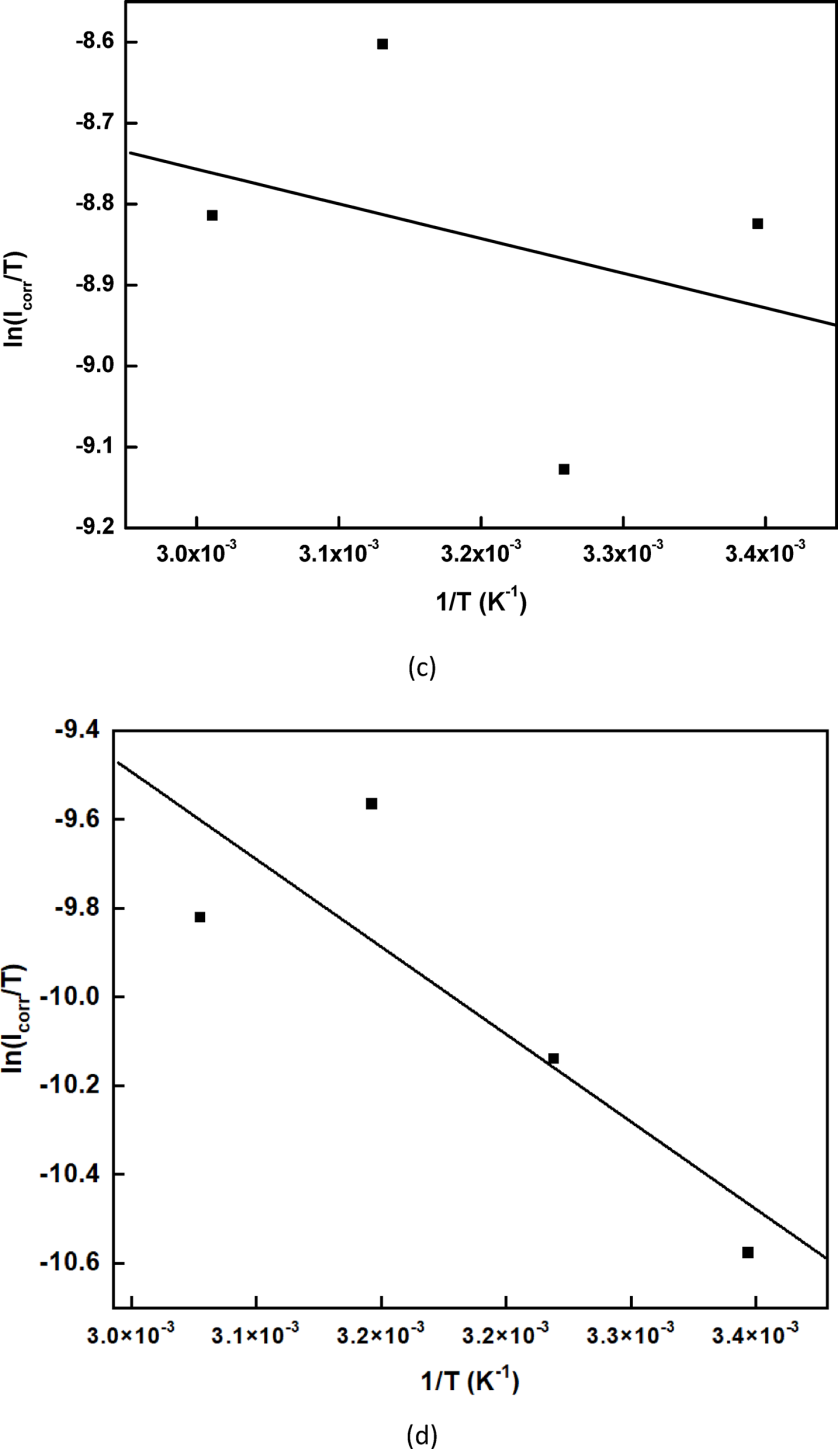
Variation of (**a**), (**b**) ln i and (**c**), (**d**) ln i/ T vs 1/T for copper in 0.5 M sulfuric acid in the absence and presence of 0.03 g/L rice straw extract.

The graphs used to calculate the activation parameter of copper in 0.5 M sulfuric acid in the absence and with 0.03 g/L of rice straw extract are displayed in Fig. 13.

The obtained activation parameter values are shown in Table 7. The swing in the activation energy values could be explained by the corrosion process’s altered mechanism when extract ingredients are present^43^.

**Table 7:** The activation parameters, obtained from polarization measurements, of copper in 0.5 M sulfuric acid in the absence and presence of 0.03 g/L rice straw extract.

	E_a_ (kJ mol^−1^)	ΔH* (kJ mol^−1^)	− ΔS* (J mol^−1^K^−1^)
Blank	8.05	4.45	256.8
Rice straw extract	26.03	23.43	205.9

The efficiency of rice straw extract is reflected in the higher values of E_a_ and ΔH* observed in its presence. The negative value of ΔS* suggests that the formation of active complex involves an association rather than a dissociation step, indicating a decrease in disorder as reactants transition to active complexes. Since the rate-determining step involves the discharge of hydrogen ions to form adsorbed hydrogen atoms on the copper surface, the presence of effective inhibitor molecules impedes this discharge process by nearly completely covering the surface^44^.

### Corrosion inhibition mechanism

**Fig. 14:**
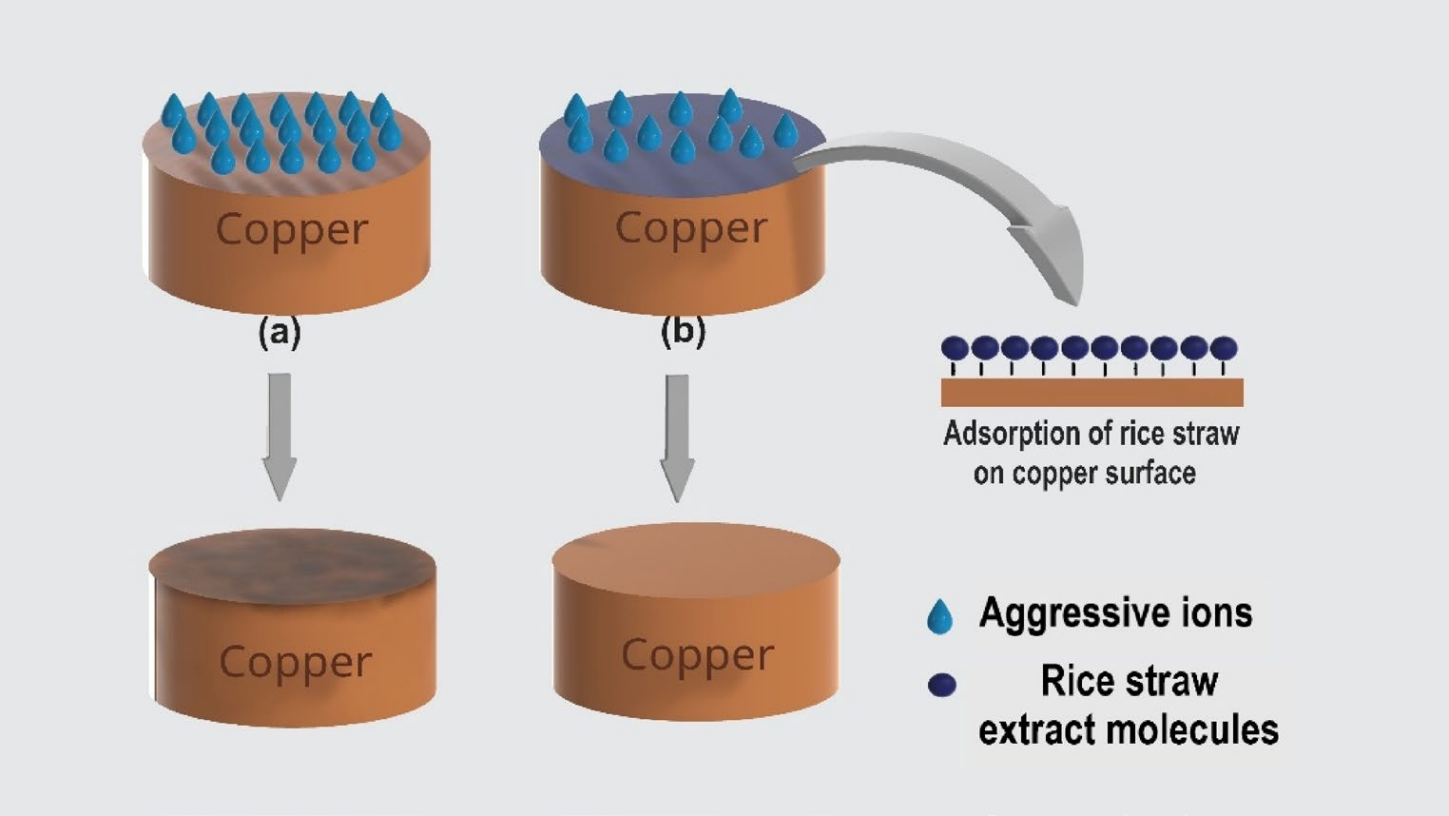
Schematic diagram of inhibition mechanism of rice straw extract for copper metal in sulfuric acid.

Figure 14 visually represents the corrosion process of copper in an acidic environment and the protective effect of rice straw extract molecules.

In the absence of any inhibitor, bare copper exposed to corrosive media undergoes direct interaction with the acidic environment, resulting in a damaged or tarnished surface, clearly indicating active corrosion. However, when rice straw extract is introduced, its bioactive molecules begin to adsorb onto the copper surface, forming a protective film that serves as a physical barrier. This barrier limits the direct contact between the copper and the corrosive agents, preserving the surface and preventing further degradation.

The inhibition mechanism is primarily attributed to the adsorption of organic compounds present in the rice straw extract onto the copper surface. These compounds create a uniform protective layer that blocks the penetration of aggressive species such as H^+^ and SO_4_^2−^ ions from the sulfuric acid medium. Electrochemical results support this, showing a decrease in double-layer capacitance (Cdl) and an increase in charge transfer resistance (Rct), both of which confirm the formation of an insulating layer that inhibits both anodic and cathodic reactions.

This schematic effectively illustrates the proposed inhibition mechanism, highlighting the role of rice straw extract in forming a passivating film that shields the metal surface from corrosion by blocking ionic interaction at the metal–electrolyte interface.

### SEM examination

**Fig. 15:**
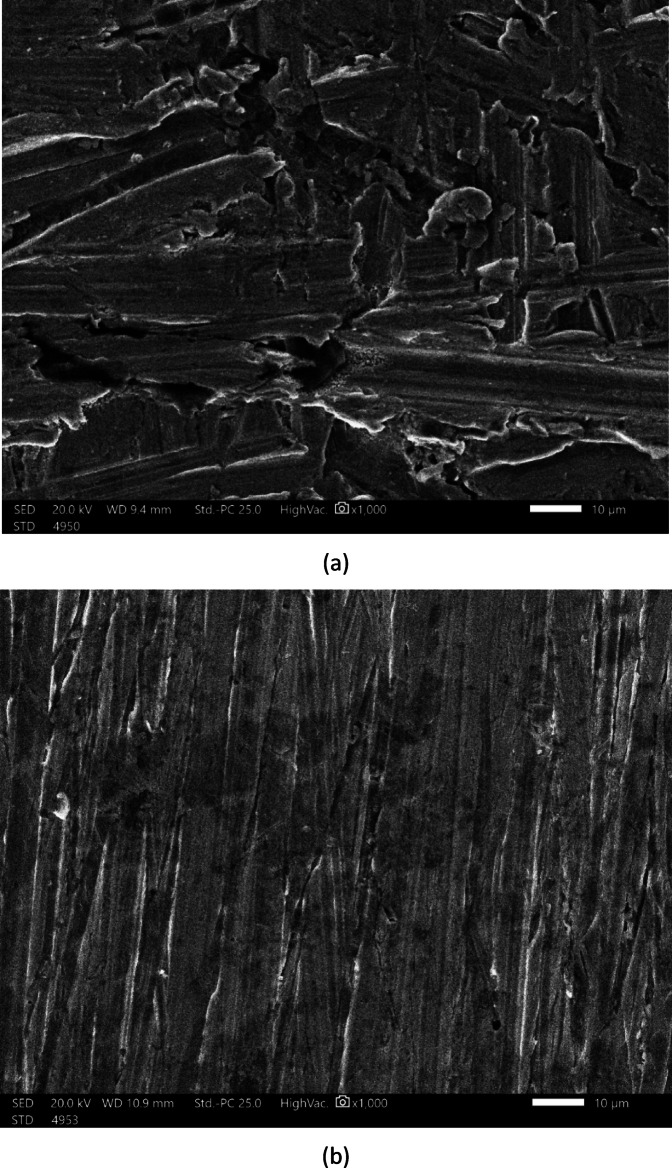
SEM micrograph of copper (**a**) in 0.5 M H_2_SO_4_ (**b**) in 0.5 M H_2_SO_4_ and 0.03 g/L rice straw extract.

We compared the SEM morphology images of copper samples that were soaked for 6 h in a 0.5 M sulfuric acid solution, both with and without the addition of 0.03 g/L of rice straw (all processes were carried out at 298 K), to further investigate the effectiveness of rice straw in mitigating copper corrosion. The results are presented in Fig. 15. In Fig. 15a, the copper sample immersed in 0.5 M H_2_SO_4_ without rice straw exhibited significant corrosion, characterized by an inconsistent and rough with numerous large, dense corrosion pits. In contrast, Fig. 15b, shows the copper surface treated with rice straw as a corrosion inhibitor, which appeared relatively smooth, aside from some surface scratches likely resulting from polishing. Additionally, there were some loose particles and microscopic holes visible on the copper’s surface, indicating signs of corrosion from the H_2_SO_4_ medium. Overall, the SEM data suggest that rice straw demonstrates some effectiveness in reducing copper corrosion.

## Conclusions

This study demonstrates that rice straw extract, a sustainable and environmentally friendly agricultural byproduct, serves as an effective green corrosion inhibitor for copper in 0.5 M H_2_SO_4_ solution. Electrochemical investigations, including potentiodynamic polarization and electrochemical impedance spectroscopy (EIS), confirmed that the extract significantly reduces the corrosion rate of copper, achieving an inhibition efficiency of approximately 91% at a concentration of 0.05 g/L and 298 °C. This performance, along with its low cost and abundance, highlights the potential of rice straw extract as a viable alternative to toxic synthetic inhibitors.

The study revealed that:

- The inhibition efficiency increases with extract concentration, indicating a dose-dependent protective behavior.
- Thermodynamic analysis confirmed a physisorption mechanism, with inhibition efficiency decreasing at elevated temperatures, consistent with physical adsorption of organic molecules on the copper surface.
- Adsorption isotherm modeling, particularly the Langmuir and kinetic–thermodynamic models, demonstrated strong binding between the inhibitor molecules and the metal surface, confirming the extract’s effectiveness.
- Surface analysis (SEM) revealed that the extract forms a protective film on the copper surface, reducing surface damage compared to uninhibited samples.

Overall, this research not only validates the corrosion inhibition performance of rice straw extract but also emphasizes the importance of utilizing agricultural waste in industrial applications. By repurposing natural biomass, this work contributes to green chemistry, waste valorization, and sustainable corrosion management strategies.

## Data Availability

The datasets used and/or analyzed during the current study are available from the corresponding author upon reasonable request.
